# Sensor game rehabilitation for children with severe motor and intellectual disabilities-medical care dependent group: feasibility case series study

**DOI:** 10.3389/fresc.2026.1833139

**Published:** 2026-06-23

**Authors:** Hiroki Annaka, Tsukasa Murakami, Takeshi Saitou, Satoko Ohmatsu, Yasuaki Kusumoto

**Affiliations:** 1 Department of Occupational Therapy, Faculty of Rehabilitation, Niigata University of Health and Welfare, Niigata, Japan; 2 Haru-bond LCC., Niigata, Japan; 3 Child Development Support Centers Haru, Niigata, Japan; 4 Digireha Inc., Tokyo, Japan; 5 Department of Physical Therapy, Fukushima Medical University School of Health Sciences, Fukushima, Japan

**Keywords:** child, disability, exergaming, medical care, parental burnout, quality of life

## Abstract

**Introduction:**

Rehabilitation for children with motor and intellectual disabilities (SMID)-medical care-dependent group (MCDG) and sub-SMID-MCDG primarily focuses on preventing deterioration and maintaining function. Rehabilitation approaches aimed at functional improvement have not yet been fully established. This study explored the feasibility of rehabilitation using sensor games for functional improvement in and sub-SMID-MCDG.

**Methods:**

This study implemented a 15-minute, once-per-week, sensor game rehabilitation program for 4 weeks. The subjects were four children with SMID-MCDG or sub-SMID-MCDG attending an after-school day service and their parents. The primary outcomes were safety of the intervention and parental feedback regarding the intervention. As secondary outcomes, the Children's Hospital of Philadelphia Infant Test of Neuromuscular Disorders, an assessment of motor function in children; the KIDSCREEN-10 parent version, an assessment of children's quality of life; and the Parental Burnout Assessment (PBA) were assessed pre- and post-intervention.

**Results:**

The 4-week sensor game rehabilitation program was safely administered to all patients. The results of the qualitative content analysis revealed that this intervention provided benefits to both children with SMID-MCDG or sub-SMID-MCDG and their parents owing to the game's high usability.

**Conclusion:**

The feasibility of sensor game rehabilitation verified in this study showed its potential to be safe and beneficial for children with SMID-MCDG or sub-SMID-MCDG and their parents. To generalize the findings of this study, further research is required to verify the effects of sensor game rehabilitation, particularly during use at home.

**Study registration:**

The study protocol was registered at the University Hospital Medical Information Network Center UMIN000055498.

## 1 Introduction

The term, children with severe motor and intellectual disabilities (SMID), refers to children with the cooccurrence of severe physical and intellectual disabilities (1). Among these children, some require ongoing medical support, including ventilators, oxygen therapy, tracheostomies, suctioning, and frequent repositioning. These children are defined as a medical care-dependent group (MCDG) or sub-MCDG (2). In Japan, the number of children classified as SMID-MCDG or sub-SMID-MCDG has increased with advances in neonatal medicine (3). Establishing adequate support systems for children with high medical dependence remains challenging (4, 5). However, the existing support systems have not fully kept pace with the needs of parents of children with SMID-MCDG or sub-SMID-MCDG, and insufficient development of rehabilitation services has also been highlighted (4, 5).

Rehabilitation interventions for children with SMID-MCDG or sub-SMID-MCDG primarily focus on preventing pressure ulcers and joint contractures, as well as providing respiratory care, because of their severe functional impairments and risk of medical complications (6–8). In current medical practice, intervention approaches aimed at improving functional abilities and expanding activity participation for these children remain in the early stages of developmental (4, 5, 9). When parents are less able to observe developmental changes in their child, this can increase the risk of parental burnout (10–12). Advancing rehabilitation for children with SMID-MCDG or sub-SMID-MCDG may therefore not only improve the child's functional abilities, but also help alleviate the psychological burden on parents.

We developed a rehabilitation program for children with SMID-MCDG and sub-SMID-MCDG using sensor games. These games detect even minimal movements, enabling children to experience enjoyment through visual changes on a screen, even in the presence of severe intellectual disabilities. The system can be easily implemented by wrapping a small sensor around one limb and connecting it to a laptop with the downloaded application. This setup allows the intervention to be conducted within a limited range without contact with medical equipment. This game has previously been validated for use in healthy individuals (13, 14). As the next step toward clinical application, we aimed to examine its feasibility for children with SMID-MCDG or sub-SMID-MCDG. Specifically, we aimed to confirm the safety of this intervention and collect feedback from parents. Additionally, this case series examines changes in motor function and quality of life in children with SMID-MCDG or sub-SMID-MCDG, as well as changes in parental burnout before and after the intervention.

## 2 Materials and methods

### 2.1 Ethics

This feasibility study was conducted in accordance with the Declaration of Helsinki, and the protocol was approved by the Institutional Review Board of Niigata University of Health and Welfare (approval no. 19405-241031). The subjects of this study were minors; therefore, their parents provided written informed consent. The study protocol was registered with the University Hospital Medical Information Network Center (UMIN000055498).

### 2.2 Subjects

This feasibility case series study was conducted on children with SMID-MCDG or sub-SMID-MCDG who utilized Haru-Garden's after-school day services. The study was conducted from May 10, 2025, to December 31, 2025, targeting children and their parents who expressed interest in the study through announcements made at after-school day services. All children who met the criteria for attending the after-school day service during the study period, along with their parents, were continuously enrolled. The inclusion criteria were as follows: 1) age: 0–18 years; 2) each category corresponding to SMID classes 1, 2, 3, and 4 of Oshima's classification based on the attending physician's diagnosis (15); 3) and a SMID-MCDG score of 10 points or higher based on the attending physician's diagnosis (2, 16). Sub-SMID-MCDG was defined by a SMID-MCDG score between 10 and 24, and SMID-MCDG was defined by a SMID-MCDG score of 25 or higher. This study did not impose any restrictions based on the underlying disease causing SMID-MCDG.

The exclusion criteria were as follows: 1) a physician's diagnosis of brain death, and 2) parents with neurological, psychiatric, or intellectual disorders that made decision-making difficult. Based on direct interviews and the medical history provided by the parent in the after-school day service records, we verified whether the parent had a history of medical conditions. In addition, children and parents who deviated from the protocol were excluded from the study.

### 2.3 Protocol

**Figure 1:**
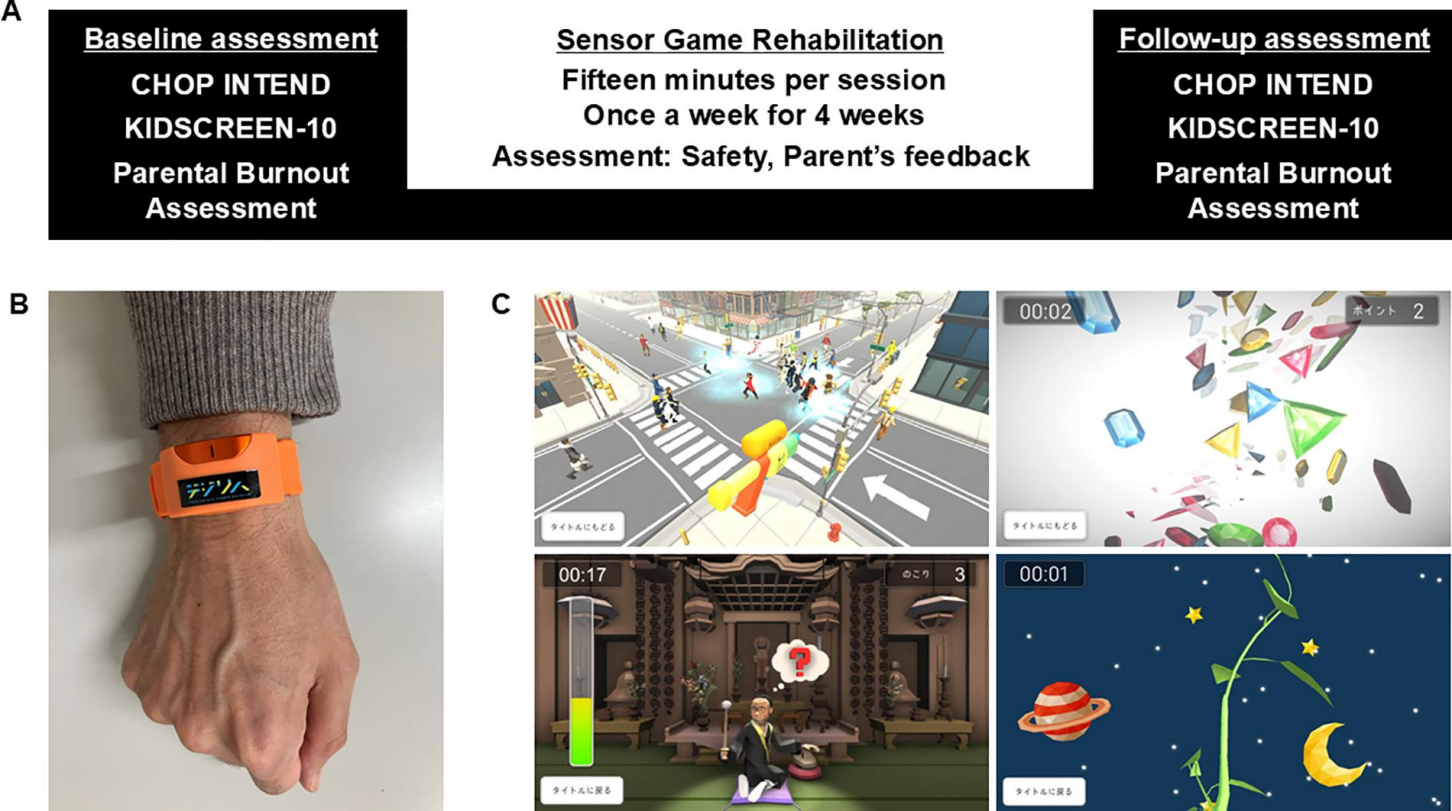
Summary of the intervention. **(A)** intervention protocol; **(B)** “Moff band” sensor used in this intervention; **(C)** The games used for the intervention. “Water Prank” is a game where sensors detect movement to shoot water at people (top left). “KIRAKIRA Jewelry” features gems that fall in sync with sensor movements (top right). “Nobi Nobi Futaba” features tree roots that grow in response to sensor movements (bottom left). “Mischief BOSE” moves its sensor when facing forward and BOSE throws objects (bottom right).

This study implemented a 15-minute, once-per-week sensor game rehabilitation program for children with SMID-MCDG and sub-SMID-MCDG during after-school day service attendance for 4 weeks (Figure 1A). The primary outcome of this study was to confirm the safety of the sensor game rehabilitation for this target group and feedback was obtained from parents. The secondary outcomes were: the Children's Hospital of Philadelphia Infant Test of Neuromuscular Disorders (CHOP INTEND), an assessment of motor function in children; the KIDSCREEN-10 parent version, an assessment of children's quality of life (QOL); and the Parental Burnout Assessment (PBA). These assessments were measured before and after intervention. No additional follow-up was conducted after the initial one-week post-intervention assessment in this study. For one week following the final intervention, facility staff monitored subjects to determine whether any acute exacerbations or medical incidents, such as intervention-related injuries, occurred. All assessments and interventions were conducted by a single researcher (H. A.), with facility staff providing support only for the CHOP INTED to enhance the scoring validity.

### 2.4 Intervention

Sensor game rehabilitation utilized the “Digireha” application (Digireha Inc., Tokyo, Japan) using an accelerometer (Moff Band; Moff Inc., Tokyo, Japan) (Figures 1B,C). Digireha is a gamification application that utilizes multiple sensors—including audio sensors, eye-tracking sensors, and accelerometers—which has demonstrated beneficial effects on neuromodulation and balance function (13, 14). This study utilized the following games: “Water Prank,” “KIRAKIRA Jewelry,” “Nobi Nobi Futaba,” and “Mischief BOSE” (Figure 1C). These games use accelerometers strapped to the body to detect movement, triggering actions on the screen such as “shooting water” or “throwing objects at Boze.” The game screen was displayed on a 17.3-inch laptop computer positioned 1 m away from the subject. The subjects were placed in either the right or left lateral recumbent position. The accelerometer was attached to either the wrist or ankle and the attachment position was changed as appropriate based on the subject's condition. Game operation was primarily performed through the subject's own movements; however, rehabilitation therapists (H.A.) provided manual support when movements against gravity proved difficult. Parents were able to observe the sensor game rehabilitation interventions.

### 2.5 Assessment

#### 2.5.1 Safety

Safety was assessed by confirming the presence or absence of acute exacerbations and medical incidents in the subjects during or after the intervention.

#### 2.5.2 Parental feedback

During the intervention, parental feedback was immediately recorded as text on a computer. This feedback-collection method was used to obtain honest, real-time, verbatim comments from parents while observing the intervention. This method is associated with a reliability equivalent to that of interview methods involving recording, compared with the interview recording (17, 18). To enhance the reliability of the records, the appropriateness of the recorded verbatim comments was confirmed with parents after the intervention. Feedback was collected by a researcher (H.A.) who had received training in qualitative content analysis.

#### 2.5.3 Children's hospital of Philadelphia infant test of neuromuscular disorders

The CHOP INTEND was used to assess motor function (19). This assessment was developed to evaluate motor function in infants with spinal muscular atrophy type I or neuromuscular disorders, and has also been used for children of a wide range of ages with severe disabilities in recent years (20–22). The CHOP INTEND, which has been validated for various diseases involving physical disabilities, can be used to assess the motor function of children with severe physical impairments (19). This tool consists of 16 items, each rated on a scale of 0–4. The total scores range from 0 to 64 points, with higher scores indicating superior motor function. Although CHOP INTEND was originally developed for infants with spinal muscular atrophy, it has been increasingly applied to assess motor function in children with various severe neuromuscular conditions (20–22). Currently, there are no validated tools available in Japan specifically designed for assessing the motor function of children with SMID-MCDG or sub-SMID-MCDG, so this study used CHOP INTEND as an alternative. The CHOP INTEND is sensitive to even slight changes in motor function, and because it was developed for infants, it is suitable for children with SMID-MCDG or sub-SMID-MCDG who have difficulty understanding instructions. CHOP INTEND was conducted following the assessment manual (19). To enhance validity, the scoring was conducted by two individuals: a researcher (H.A.) who was trained in the assessment method and a facility staff member who provided routine care to the children. The researcher (H.A.) assessed the children, while facility staff observed the scene and scored. The researcher (H.A.), who had received training in the assessment method, instructed other assessors on the procedures and verified the accuracy of their scores. After the assessment, the two assessors immediately compared their scores. In this study, it was established that items with non-matching assessment scores would be reassessed; however, no discrepancies were identified in any of the assessments. All assessments were conducted while the child was awake.

#### 2.5.4 KIDSCREEN-10 parent version

The KIDSCREEN-10 parent version was used to assess the QOL. This tool assesses a child's QOL in the physical, mental, and social domains (23, 24). The 10 items are rated on a 5-point Likert scale ranging from “Never” (1) to “Always” (5), with higher values indicating higher QOL. Unlike the child version, the parent version uses a proxy assessment, making it suitable for a wide range of age groups (23, 25). The researcher (H.A.) followed the assessment manual and asked the parents to complete the questionnaire (24). The parents completed the questionnaire following the instructions. Scoring was performed by researchers (H.A) following the scoring manual.

#### 2.5.5 Parental burnout assessment

Parental burnout was measured using the PBA. This 23-item questionnaire assesses the following four core symptoms of parental burnout: emotional exhaustion (nine items), contrast with previous parental self (six items), loss of pleasure in one's parental role (five items), and emotional distancing from one's children (three items) (26). Each item is rated on a 7-point frequency scale ranging from 0 to 6, with a higher total score indicating greater parental burnout. The researcher (H.A.) explained the procedure to the parents according to the manual (26). After listening to the researcher's (H. A) explanation, the parents filled out each item. Scoring was performed by the researchers (H.A) in accordance with the scoring manual.

### 2.6 Analysis

Feedback from parents was analyzed using an inductive approach based on the content analysis methodology of Graneheim and Landman. To gain a deep understanding of the data and develop general concepts, the verbatim transcripts were carefully read multiple times (27). During the analysis process, interpretations were refined by continuously comparing the data as a whole with individual statements. Sentences containing content related to sensor game rehabilitation were identified as units of meaning. Each meaning units was concisely summarized while retaining its context, and a code was assigned reflecting its content. Care was taken to remain faithful to the original meaning during coding. Sub-categories were formed based on code similarity and content validity and further refined through repeated cross-checking against the data. Categories were derived through comparisons between subcategories.

The analysis was led by the first author (H.A.), who conducted a close reading of the verbatim transcripts, extracted meaning units, performed initial coding, and created subcategories. Subsequently, the second author (T.M.) critically reviewed these analytical processes and results, examining the validity of the codes and subcategories. Both authors engaged in repeated discussions regarding the coding and categorization processes to confirm the consistency and validity of their interpretations. In particular, when determining the categories, the first and second authors carefully discussed the matter based on the two axes—horizontal and vertical—in the dataset, prior to member checking. When discrepancies arose, they revisited the original text and continued discussions until a consensus was reached.

To enhance the trustworthiness of the analysis results, all researchers verified the member checking. Data saturation was also determined through member checking. When differences of opinion arose among the researchers, they were discussed through iterative exchanges among the authors, and consensus was reached by integrating their perspectives. Furthermore, to ensure transparency in the analysis, interpretations, and decision-making at each stage were documented. Additionally, the lead author engaged in continuous self-reflection throughout the analysis, striving to ensure that the researchers' preconceptions or interpretive biases did not influence the results.

The CHOP INTEND, KIDSCREEN-10 parent version, and PBA scores were presented descriptively.

## 3 Results

### 3.1 Characteristics of children and parents

Among the eight families with children attending after-school day services, one family was excluded from the brain death determination. Two of the seven pairs meeting the inclusion criteria declined participation, leaving five parent-child pairs to participate. In addition, one child and their parent withdrew from the study prior to the pre-assessment owing to an acute exacerbation. Table 1 presents the characteristics of the children and their parents. Three children were classified as SMID-MCDG and one was sub-SMID-MCDG. Case 3 required manual assistance from a therapist because of difficulty in performing voluntary movements against gravity. Children other than case 3 had at least one limb capable of voluntary movement against gravity.

**Table 1:** Characteristics of children and parents.

Variable	Case 1	Case 2	Case 3	Case 4
Child				
Age (years)	10	5	8	7
Sex	Male	Female	Male	Female
Diagnosis	Hereditary dystonia	Cerebral palsy with epilepsy	West syndrome	Larsen syndrome
SMID-MCDG score	21	30	32	37
Medical care	Tracheotomy Frequent suction of airway secretions Gastrostomy tube feeding	Tracheotomy Frequent suction of airway secretions Frequent use of nebulizer Duodenal gastric feeding tube Frequent position changes	Tracheotomy Oxygen inhalation Frequent suction of airway secretions Frequent use of nebulizer Duodenal gastric feeding tube Frequent position changes	Ventilator Tracheotomy Frequent suction of airway secretions Frequent use of nebulizer Duodenal gastric feeding tube Frequent position changes
Oshima’s classification	1	1	1	1
GMFCS E & R	V	V	V	V
Parent				
Age (years)	51	33	38	39
Sex	Female	Male	Female	Female
Education	High school graduate	Junior college graduate	High school graduation	Junior college graduate
Partner	Yes	No	Yes	Yes
Employment	No	No	Yes	No
Number of children	2	4	2	2
Daily sleep time (hours)	7	5	6.5	5.5

SMID-MCDG, severe motor and intellectual disabilities-medical care-dependent group; GMFCS E & R, gross motor function classification system extended & revised.

### 3.2 Safety

No injuries or medical accidents occurred during the implementation of the sensor game rehabilitation intervention, and none were observed during the one-week follow-up period after the final intervention. Preparation for the intervention was completed by one therapist within 5 min. The intervention had no impact on ventilator tubing. Synchronization between the computer and sensors, along with the application launch, proceeded smoothly in all cases.

### 3.3 Parental feedback

Table 2 lists the meaning units, codes, subcategories, and categories generated from parental feedback regarding the sensor game rehabilitation. Categories were divided into “Intervention effect” and “Game design.”

**Table 2:** Meaning units, codes, subcategories, and categories generated from parental feedback regarding sensor-game rehabilitation.

Category	Sub-category	Code	Meaning unit	Parent’s feedback
Intervention effect	Effect on children	Child’s immersion in games	I learned for the first time that child can play with such concentration	*“I’ve never seen him play with intense focus before.”* (case 1)
		Improving child’s gross motor skills	Child’s motor skills improved	*“Since starting the game, I feel like my child’s head and hand movements have become more active.”* (case 2)
		Reduction in childcare burden	It’s now easier to support the child while she sits	*“It’s become easier to help children sit down.”* (case 2)
		Child’s enjoyment	It became an enjoyment for the child	*“The child was enjoying himself.”* (case 2)
			It became an enjoyment for the child	*“The child seemed to be having fun.”* (case 4)
	Effect on parent	Parental joy	I was happy that my child played well	*“He can’t play well with other toys, but I was happy he could play with this one.”* (case 3)
		Enhancing parental understanding of child	I was able to understand the child’s abilities	*“Until now, I wasn’t sure if this child was moving consciously, but seeing them stare at the game screen and move their body in sync with the screen’s timing, I realized they were moving consciously, and I was delighted.”* (case 3)
Game design	Game usability	Variety of games	The game selection was extensive	*“There were many different kinds of games, so the child never got bored playing.”* (case 2)
		Game suitability for child	This rehabilitation was suitable for child	*“This was the best rehabilitation he had ever received.”* (case 3)
			This game was suitable for child	*“It was the most energetically waving its arms out of all the toys she have played with so far.”* (case 4)
		The uniqueness of the game	The game was unique	*“The game’s content and title looked interesting.” (*case 4)

#### 3.3.1 Intervention effect

Based on parental feedback, the sensor-game rehabilitation was confirmed to be effective for both children and parents. Effects on the children included improvements in physical function and enjoyment. Effects on parents included providing opportunities for joy and promoting an understanding of their child's abilities.

#### 3.3.2 Game design

The sensor game used in the intervention was confirmed to be a highly usable game design for SMID-MCDG and sub-SMID-MCDG children. Parent's expectations indicated that the game content was of good quality, specifically the richness and originality of the games was affirmed, as well as its suitability for children.

### 3.4 Secondary outcomes

Table 3 shows the pre- and post-intervention scores for the CHOP INTEND, KIDSCREEN-10 parent version, and PBA. The CHOP INTEND scores improved in three children (Case 1 increased by 2 points, Case 2 by 9 points, and Case 4 by 3 points), whereas one child showed no change (Case 3). The KIDSCREEN-10 parent version scores improved for two children (Case 2 increased by 10 points and Case 3 by 1 point), remained no change for one child (Case 1), and decreased for one child (case 4 decreased by 4 points). Case 4 showed a decline in scores, with a decrease in items reflecting parental involvement with their child. Parental burnout was reduced in two parents (Case 3 decreased by 4 points and Case 4 by 18 points), whereas the other two parents showed no change (Cases 1 and 2).

**Table 3:** Pre- and post-intervention results for Children’s Hospital of Philadelphia Infant Test of Neuromuscular Disorders, KIDSCREEN-10 parent version, and Parental Burnout Assessment.

Case	Case 1		Case 2		Case 3		Case 4	
	Pre	Post	Pre	Post	Pre	Post	Pre	Post
Children’s Hospital of Philadelphia Infant Test of Neuromuscular Disorders (score)	38	41	22	31	4	4	11	14
KIDSCREEN-10 parent version (score)	50	50	34	44	27	28	49	45
Parental Burnout Assessment (score)	0	0	10	10	6	2	25	7

## 4 Discussion

**Figure 2:**
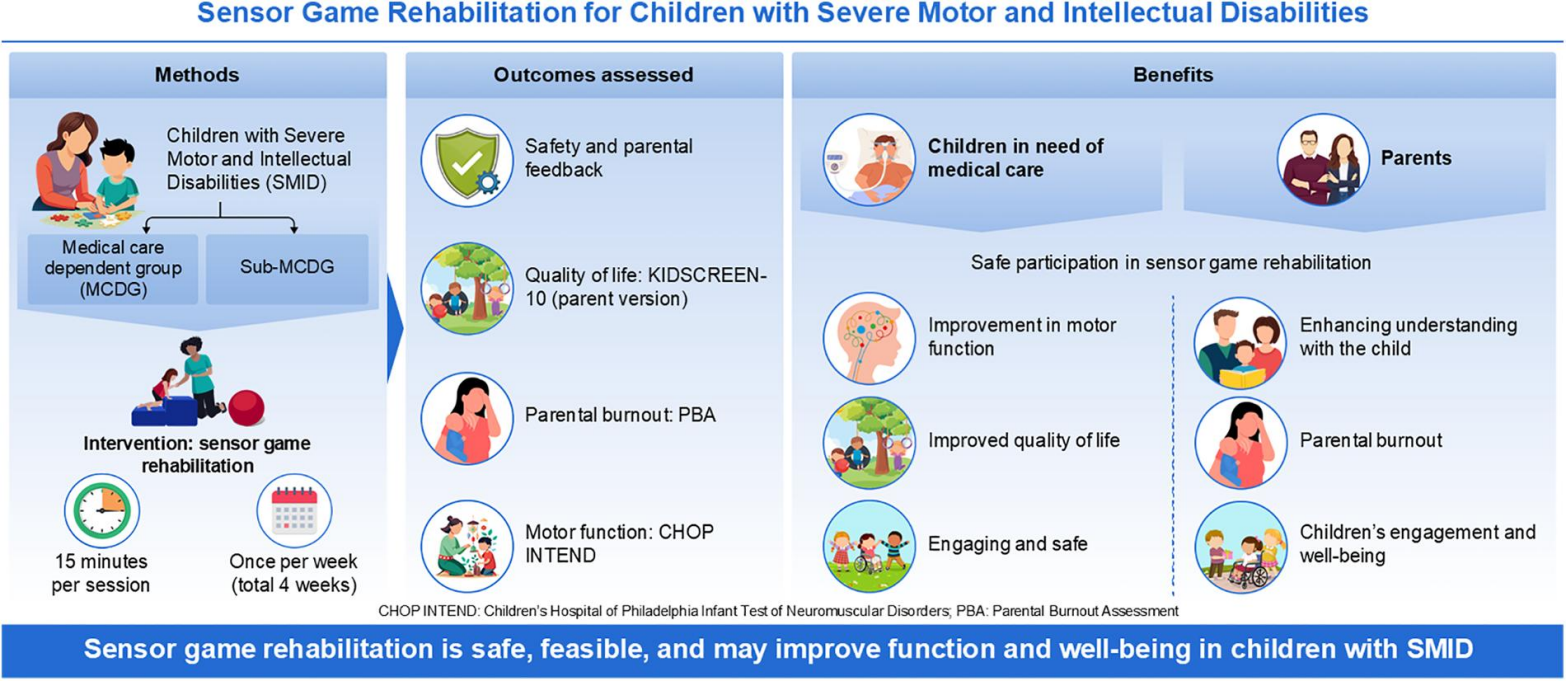
Summary of results.

This study verified the feasibility of sensor game rehabilitation in children with SMID-MCDG and sub-SMID-MCDG (Figure 2). Most rehabilitation programs for these children involve interventions to prevent acute exacerbations, pressure ulcers, and joint contractures (6–8). However, in recent years, improving children's abilities and supporting parents' emotional well-being have become challenging (5, 6–8). The development of rehabilitation programs to support children with SMID-MCDG or sub-SMID-MCDG and their parents is an urgent priority, but remains insufficient (5, 6–8).

Qualitative content analysis showed that sensor game rehabilitation was effective in improving children's functional abilities, providing opportunities for enjoyment and increasing parental joy. Technology helps children and adults with SMID expand their range of activities, enabling them to deepen their bond with their parents through these acquired activities (28–30). Furthermore, through technology-based activities, parents can better understand their child's capabilities and deepen the bond of affection (28). The findings of these previous studies are similar to those of the present study targeting SMID-MCDG or sub-SMID-MCDG children. However, a significant difference from previous studies was that a single sensor and application could manage all cases. Children or individuals with SMID require customized technological adaptations (28–30). This is because the specific body parts that can be moved differ slightly from person to person. Sensor games using accelerometers, such as those employed in this intervention, can be operated by individuals with physical disabilities by simply enabling detection of slight movements and adjusting the application settings (31, 32). Additionally, this sensor game offers the simplicity of wrapping an accelerometer around a child's limbs, along with the advantage of being able to be performed in a minimal space, even when medical equipment such as ventilators are present (33–35). However, it should be noted that these results are based on data collected from motivated parents who participated in the study and may have been influenced by the heightened emotions resulting from exposure to new technology over a short period of one month. Furthermore, the parents themselves are not configuring the sensor game or assisting their children. While there are matters that require ongoing discussions, sensor game rehabilitation holds potential as a new optimal rehabilitation method for children with SMID-MCDG or sub-SMID-MCDG.

While assessing feasibility, changes in motor function were observed in three children. Children with SMID tend to have limited experience with voluntary movement, but using sensor-based games can boost their motivation and encourage them to move (36–38). In this study, changes were also observed in children's QOL and parental burnout. These changes in secondary outcomes were limited to observations of differences between the pre- and post-assessment, and it remains unclear whether they represent a long-term phenomenon. Furthermore, based on the results of this study, the effectiveness of this intervention may vary depending on the child's characteristics, such as severity, abilities, and underlying medical conditions. In the next phase, it will be necessary to evaluate the effectiveness of this sensor game rehabilitation on children's motor function and QOL, as well as on parental burnout.

This study has methodological limitations that restrict its findings to feasibility without enabling conclusions about effectiveness. First, the outcomes of this study are subject to potential bias due to the subjectivity of researchers and respondents. As this study did not employ a blinded design, the results may have been influenced by response bias, as parents may have avoided providing negative responses in their feedback and survey answers. Although we have taken the necessary measures to enhance reliability and validity, the possibility of residual measurement biases remains. In particular, despite efforts to minimize subjective bias among researchers in the qualitative content analysis, since the parental feedback was collected by a single researcher, the potential for confirmation bias cannot be completely excluded. Furthermore, while the CHOP INTEND is a reliable and valid tool for assessing children with severe physical disabilities, it has not been specifically validated for the study population; therefore, the possibility of measurement bias remains. Since the sensitivity and construct validity have not yet been verified, the changes observed in this study should be interpreted with caution. There are currently no tools available to assess motor function in children with SMID-MCDG, and CHOP INTEND may serve as a potential alternative. Future research is needed to validate the validity of CHOP INTEND for this population. Second, no statistical analysis was performed to verify changes in secondary outcomes due to the exploratory nature of this feasibility study. Third, the absence of a control group and lack of blinding in outcome assessments may have introduced bias. Fourth, the short intervention period of only 4 weeks with weekly 15-minute sessions may not have been sufficient to observe substantial changes. As this feasibility study focused on children receiving medical care who had difficulty communicating, the frequency of interventions was kept to a minimum to avoid causing them undue fatigue. Moreover, given that this study confirmed the safety of the intervention, the next phase should examine the appropriate frequency of intervention. Fifth, this single-center study examined the effects of therapist-led intervention within the facility, which may limit external validity. One reason is the small sample size of only four pairs of children and parents. Limiting recruitment to a specific population may have introduced selection bias. The second reason is that no statistical analysis was performed to verify changes in secondary outcomes. Additionally, as this study was not randomized or controlled, changes in secondary outcomes should be discussed in the next phase. The third reason is that this study examines the effects of therapist intervention within the facility. To generalize this study findings, it is necessary to verify the effectiveness of implementing the program solely with the child and parents in the home. In the next phase, we need to verify these limitations with a view toward generalization, taking these challenges into account.

## 5 Conclusion

The feasibility of sensor game rehabilitation verified in this study showed its potential to be safe and beneficial for children with SMID-MCDG or sub-SMID-MCDG and their parents. Owing to the limitations in the research methodology, it is difficult to substantiate the effectiveness of this intervention for these children and their parents; however, it holds promise as a new rehabilitation strategy. Further research is needed to verify the effects of sensor game rehabilitation in children with SMID-MCDG and sub-SMID-MCDG and their parents.

## Data Availability

The original contributions presented in the study are included in the article/Supplementary Material, further inquiries can be directed to the corresponding author.
